# Study on small molecular organic compounds pyrolysed from rubber seed oil and its sodium soap

**DOI:** 10.1186/s40064-016-1955-5

**Published:** 2016-03-12

**Authors:** T. L. D. Fernando, M. A. B. Prashantha, A. D. U. S. Amarasinghe

**Affiliations:** Department of Chemistry, University of Sri Jayawardenepura, Nugegoda, Sri Lanka; Department of Chemical and Process Engineering, University of Moratuwa, Moratuwa, Sri Lanka

**Keywords:** Pyrolysis, Rubber seed oil, Renewable, Organic chemical precursors

## Abstract

Rubber seed oil (RSO) and its sodium soap were pyrolysed in a batch reactor to obtain low molar mass organic substances. The pyrolitic oil of RSO was redistilled and the distillates were characterized by GC–MS and FTIR. Density, acid value, saponification value and ester values were also measured according to the ASTM standard methods. A similar analysis was done for samples taken out at different time intervals from the reaction mixture. Industrially important low molar mass alkanes, alkenes, aromatics, cyclic compounds and carboxylic acids were identified in the pyrolysis process of rubber seed oil. However, pyrolysis of the sodium soap of rubber seed oil gave a mixture of hydrocarbons in the range of C_14_–C_17_ and hence it has more applications as a fuel.

## Background

Organic chemical precursors have significant utility in the petroleum industry. Such petrochemicals provide useful organic precursors for a wide range of industries such as plastics, synthetic rubber, synthetic clothing, pharmaceuticals, solvents, lubricants, detergents, adhesives, surface coatings, agrochemicals, artificial colours and flavours. However, the origin of petrochemicals is fossil fuels which are a non-renewable resource currently in the declining phase of use (Hubbert 1956; Mohsen and Nahid 2015).

Pyrolysis is the thermal cracking of high molecular organic substances into small molecules in the absence of oxygen. Bio mass pyrolysis is a proven method of producing a broad mixture of small molecular organic compounds (SMOC; Bridgwater 2003) which can be isolated and treated as alternatives for renewable organic chemical precursors. The petrochemical industry is also based on seven different building blocks, namely, ethylene, propylene, butane, butylene, butadiene, syn gas and BTX (benzene, toluene and xylene), and all organic chemicals can be derived from these building blocks (Brown 2003). Thus, the pyrolysis of triglyceride materials is a convenient, realistic and economical method of producing SMOC and thereby to producing renewable organic chemical precursors.

A range of SMOC have been reported as being derived from the pyrolysis of different types of triglyceride materials. Soybean oil and Castor oil in a batch system at a temperature of 350 °C yielded alkanes (C_11_–C_15_), alkenes (C_11_–C_15_), carboxylic acids (C_7_–C_11_) and aldehydes (C_11_–C_15_) and alkanes, alkenes, aldehydes, ketones of C_15_–C_16_ and carboxylic acids of C_11_–C_12_ with a higher portion of oxygenated products, respectively (Lima et al. 2004). The pyrolysis of palm oil at 375 °C yielded saturated fatty acids, hydrocarbons and some oxygenated products (Twaiq et al. 2004). Similarly, the pyrolysis of canola oil in a flow reactor at 400 °C produced alkenes and aromatics (Idem et al. 1996). Also, the pyrolysis of the soap of palm and rapeseed oils at 750 °C gave saturated hydrocarbons, while castor seed oil gave ketones and phenols (Hanna 2011).

*Hevea brasiliensis* (rubber) is the source of natural rubber latex which can be used to produce surgical gloves, tires, conveyor belts, windshield wipers, toys and various other rubber goods. Rubber is cultivated as large plantations, in many countries such as Sri Lanka, India, Indonesia, Vietnam, China and Congo Free State in Africa. Rubber seeds were originally considered as a waste product but now they are used to feed livestock (Abdullah and Salimon 2009). Rubber seed contains a relatively higher oil content of 40–50 % (Junaid et al. 2014) and the rubber seed oil (RSO) was found to have properties similar to soya bean oil and linseed oil which are widely used in industrial applications. Therefore, RSO has now become an important raw material for many industries; as semidrying oil in the paint industry (Joseph et al. 2004), in soap manufacturing (Ohikhena 2006), as anti-malaria oil in medicines (Thomas et al. 1998) and as a core binder for factice preparation in engineering (Fernando 1971).

RSO may become significant as a raw material in the pyrolysis process due to its relatively high oil content, geological distribution over a wide area, free availability and non-edible nature. The identification of the potential of RSO to produce SMOC will be an added advantage for rubber plantations. The percentage of unsaturated fatty acids is important for obtaining different SMOC since saturated fatty acids are especially suitable for obtaining linear hydrocarbons (Knothe et al. 1997). The total content of unsaturated fatty acids in rubber seed oil is relatively higher than those of palm and coconut oils but is comparable with that of soybean oil. The fatty acid composition of soybean oil is Linoleic 54.51 %, Oleic 22.98 %, Stearic 4.09 %, Palmitic 10.58 %, and Linolenic 7.23 % (Farzana et al. 2011) whereas the fatty acid composition of RSO is Linoleic 39.60 %, Oleic 24.60 %, stearic 8.70 %, Palmitic 0.2 % and Linolenic 16.30 % (Junaid et al. 2014). The pyrolysis of soybean oil has produced olefins, paraffins, carboxylic acids and aldehydes of C_10_–C_18_ (Junming et al. 2009) and hence, RSO also has a potential to become a good renewable source for pyrolysis. Thus the aim of this study is to identify the SMOC obtained by the pyrolysis process of RSO and sodium soap of RSO as an alternative renewable source for petrochemicals.

## Methods

The RSO was extracted using solvent extraction with a standard Soxhlet apparatus using n-hexane as the solvent. The acid value (ASTM D 664), saponification value (ASTM D 1926) and density (ASTM D 4052) were determined. The acid value indicates the amount (mg) of potassium hydroxide required to neutralize free fatty acids in 1 g of fatty oil, while the saponification value measures the total amount (mg) of potassium hydroxide required to cleave ester bonds and to neutralize free fatty acids in 1 g of fatty oil. The ester value can be calculated as the difference between the SAP and acid values.

**Fig. 1:**
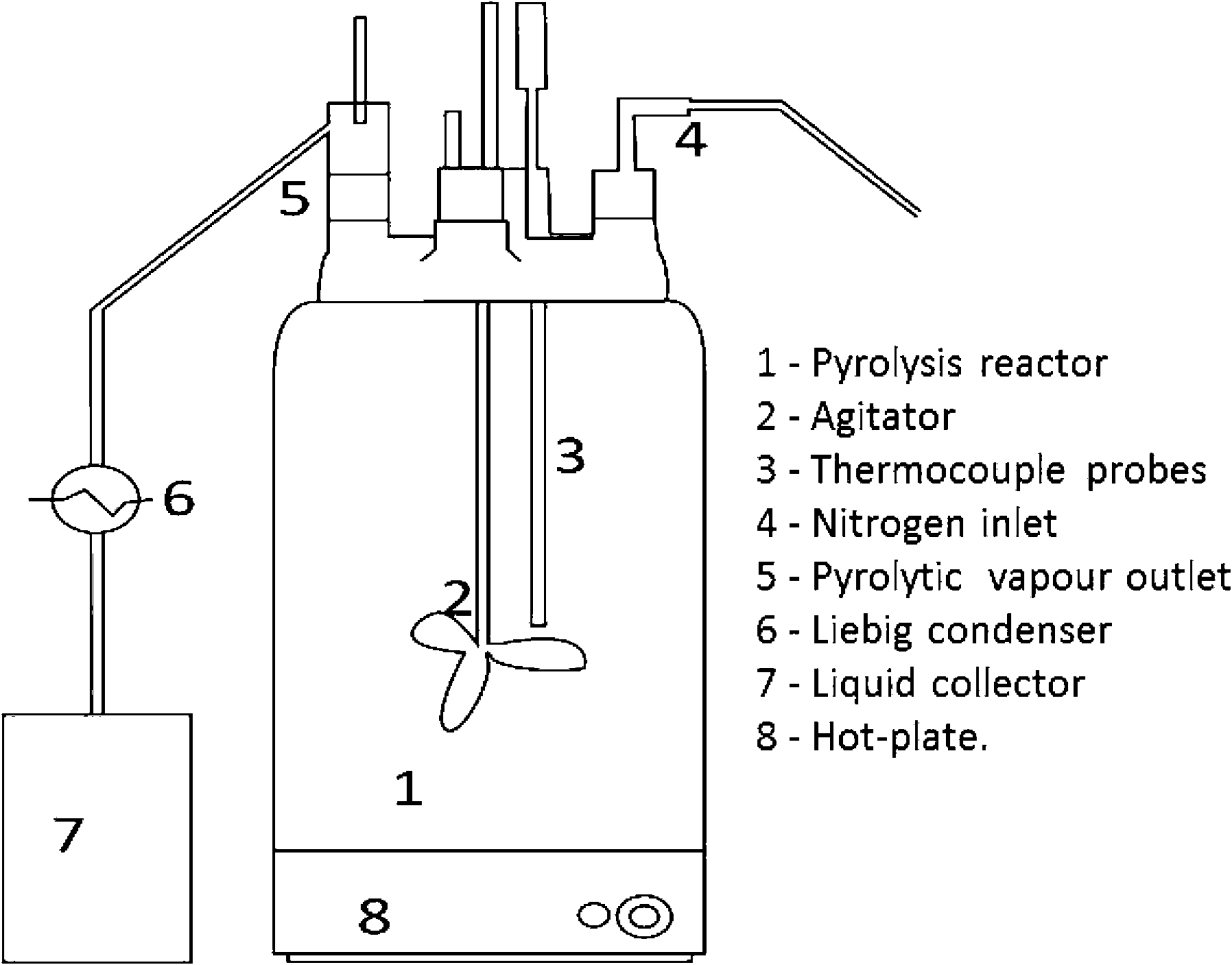
Stainless steel batch reactor unit used for pyrolysis

The pyrolysis of RSO was carried out within an inert nitrogen atmosphere using a 500 ml volume of RSO in a five neck stainless steel batch reactor unit equipped with two thermocouples for the liquid and vapour phases, a nitrogen gas inlet, a stoppered sample port, a Liebig condenser and a mechanical agitator as shown in Fig. 1. The temperature of the liquid phase was continuously monitored at different time intervals to identify the time–temperature profile, which is important for determining the heating rates and temperature variations in the pyrolysis process. The rotating speed of the mechanical agitator was set to 400 rpm to homogenize the mixture and to stabilize the temperature. The sample port was used to collect samples from the reaction mixture at different time intervals to analyse changes in acid value, saponification value, ester value and density. The pyrolitic vapour was condensed and collected as pyrolytic oil. The pyrolysis process was continued until the total yield of pyrolitic oil was collected.

The organic phase of the pyrolitic oil of RSO was separated from the aqueous phase using a separatory funnel and then redistilled to collect equal 30 ml fractions of distillates. The corresponding range of distillation temperatures (DT) were recorded for each such fraction. Four different distillates, D1, D2, D3 and D4, were obtained corresponding to ranges of temperatures, DT < 80 °C, 80 °C < DT < 120 °C, 120 °C < DT < 140 °C and 140 °C < DT < 160 °C, respectively. All the distilled fractions were analysed by GC–MS and FTIR. The physicochemical properties such as acid value, SAP value and density were determined according to the ASTM standards. The ester value was calculated by the difference between the saponification and acid values. GC–MS analysis was carried out on an Agilent 5975C chromatograph coupled with an MSD-EC detector and equipped with a HP-SMS 5 % Phenyl methylsiloxane non polar capillary column of 30.00 m length, 250 μm diameter and 0.25 μm film thickness. High purity Helium was used as the inert carrier gas. The inlet temperature was kept at 50 °C for 5 min holding time and the temperature was increased by 10 °C min^−1^ up to 250 °C and kept at 250 °C for a further 10 min. The inlet heater was set to 100 °C and a pressure of 12.675 psi and the total flow rate was 154.5 ml min^−1^. Peak assignment was done with the help of a NIST08 database with more than 90 % similarity.

The Na-soap of RSO was prepared by the hot process (Asuquo et al. 2012) and it was used as a feed stock for pyrolysis. The time and temperature data in the reaction mixture were recorded as was done in the case of the pyrolysis process of the RSO. The organic phase of pyrolytic oil obtained from Na-soap of RSO was analysed by GC–MS according to the same time–temperature program.

## Results and discussion

### Time–temperature profiles of the pyrolysis process of RSO and Na-soap of RSO

**Fig. 2:**
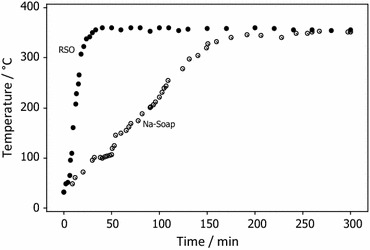
Time–temperature profiles of pyrolysis of RSO and its Na-soap

The pyrolysis experiment was carried out with continuous heating until the condensation of the pyrolytic oil was achieved. The observed time–temperature profile of the pyrolysis of RSO and Na-soap of RSO is shown in Fig. 2. The temperature of the pyrolysis process of the RSO system rose to 350 °C during the first 50 min at an average rate of 12.60 °C min^−1^. The increase in temperature of the RSO medium was more rapid than of its Na-soap in solid state. This can be mainly attributed to the higher tendency for molecular movements in the liquid than the solid state. Even though the heating was continued, the temperature of the reaction mixture became almost constant after reaching 350 °C and was found to remain constant under the given experimental conditions until the pyrolysis reaction was completed.

The temperature gradually rose up to 100 °C at an average rate of 8.40 °C min^−1^ during the pyrolysis process of the Na-soap of RSO. Then, the temperature remained constant at 100 °C for about 20 min which may have been due to the evaporation of entrapped water within the mass of soap. A volume expansion was also observed for the Na-soap of RSO at around 100 °C which confirms the evaporation of bound water. The temperature rose further at an average rate of 8.60 °C min^−1^ up to 350 °C and remained constant until the pyrolysis reaction was completed.

### Analysis of physicochemical changes in reaction mixture during pyrolysis of RSO

**Fig. 3:**
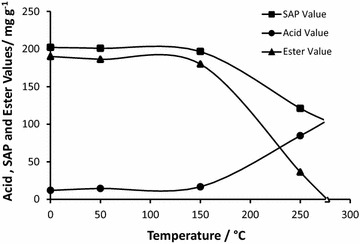
Variation of acid, SAP and ester values in the reaction mixture during the pyrolysis process of RSO

Samples were taken at different temperature levels from the reaction mixture to examine the end point of ester bond cleavage in triglycerides of RSO by analysing the acid, SAP and ester values. Figure 3 shows the variation of acid, ester and saponification values in the reaction mixture of RSO at different time intervals during the heating period.

**Fig. 4:**
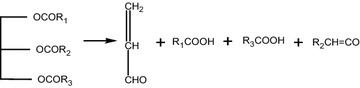
The initial step of pyrolysis reaction

The trend in acid value shows a very slight increase during the initial heating period until it reached 150 °C and a significant increase after reaching 150 °C. Similarly, the SAP value decreased very slightly until it reached 150 °C and fell very rapidly after reaching 150 °C, indicating that the thermal cleavage of triglycerides (TG) into free fatty acids (FFA) became significant as the temperature exceeded 150 °C. Extrapolation of the graphs beyond 250 °C indicates the end point of ester bonds in triglycerides at a temperature around 275 °C. The observed trends of acid and SAP values also agree with the reaction proposed in literature (Chang and wan 1947) where free fatty acids, propenal (acrolein) and ketenes, are formed as the initial step of pyrolysis, and is shown in Fig. 4.

The densities at room temperature were also tested for the samples taken from the reaction mixture at 30, 50, 150 and 250 °C and the values were found to be 919.4, 922.2, 925.6 and 932.7 kg m^−3^ respectively. The slight increase in density indicates that the intermolecular interactions became significant with the increase of free fatty acids during the heating period.

### Yield of different phases in the pyrolysis process

The vapour phase temperature was found to be about 250 °C in both RSO and Na-soap samples when the temperature of the reaction medium had achieved the steady state of 350 °C. In the previous studies, pyrolysis of soya bean, palm and caster oils were carried out at the temperature of 350 °C (Lima et al. 2004) while the pyrolysis of canola oil was carried out in a flow reactor unit in the temperature range of 350–400 °C (Idem et al. 1996).

**Fig. 5:**
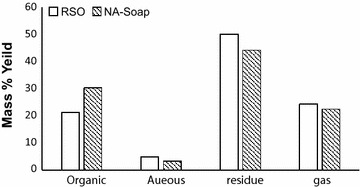
Mass percentage yields of different fractions with respect to the feed mass

The pyrolitic oil was collected through the condenser and it consisted of organic and aqueous phases. A highly viscous black coloured liquid residue remained in the reactor vessel after the pyrolysis process of RSO whereas a black coloured solid residue remained at the end of the pyrolysis process of Na-soap. The yield percentages of the organic and aqueous phases of pyrolitic oil, liquid and solid residues and non-condensed gaseous products were calculated with respect to the input mass and are shown in Fig. 5. The non-condensed gaseous products led to mass loss (Andre et al. 2010) and it was calculated by considering the mass balance. The non-condensed gas was expected to be a mixture of C_1_–C_5_ hydrocarbons and CO_2_ gas according to the reaction mechanism proposed by Idem et al. (1996).

The yield of organic phase is higher in pyrolytic oil of Na-soap of RSO (30 %) than that of RSO (21 %). This may be due to the compact micelles arrangement of soap molecules which facilitates efficient molecular collisions, the effect of charged particles (sodium ion) and the ionic strength of the soap. The yield percentages of the pyrolysis of soybean soap stock were previously reported as 29 % for gaseous products, 22 % for the aqueous phase of pyrolitic oil, 31 % for the organic phase of pyrolitic oil and 18 % for the solid residue (Andre et al. 2010). The yield percentage of the organic phase in pyrolitic oil of RSO soap was comparable to that of pyrolitic oil of soybean soap. However, the aqueous phase of pyrolitic oil (3 %) was considerably low and the residual waste (44 %) was significantly high in RSO soap compared to soybean soap.

### Analysis of distillation components of the organic phase of pyrolytic oil

**Fig. 6:**
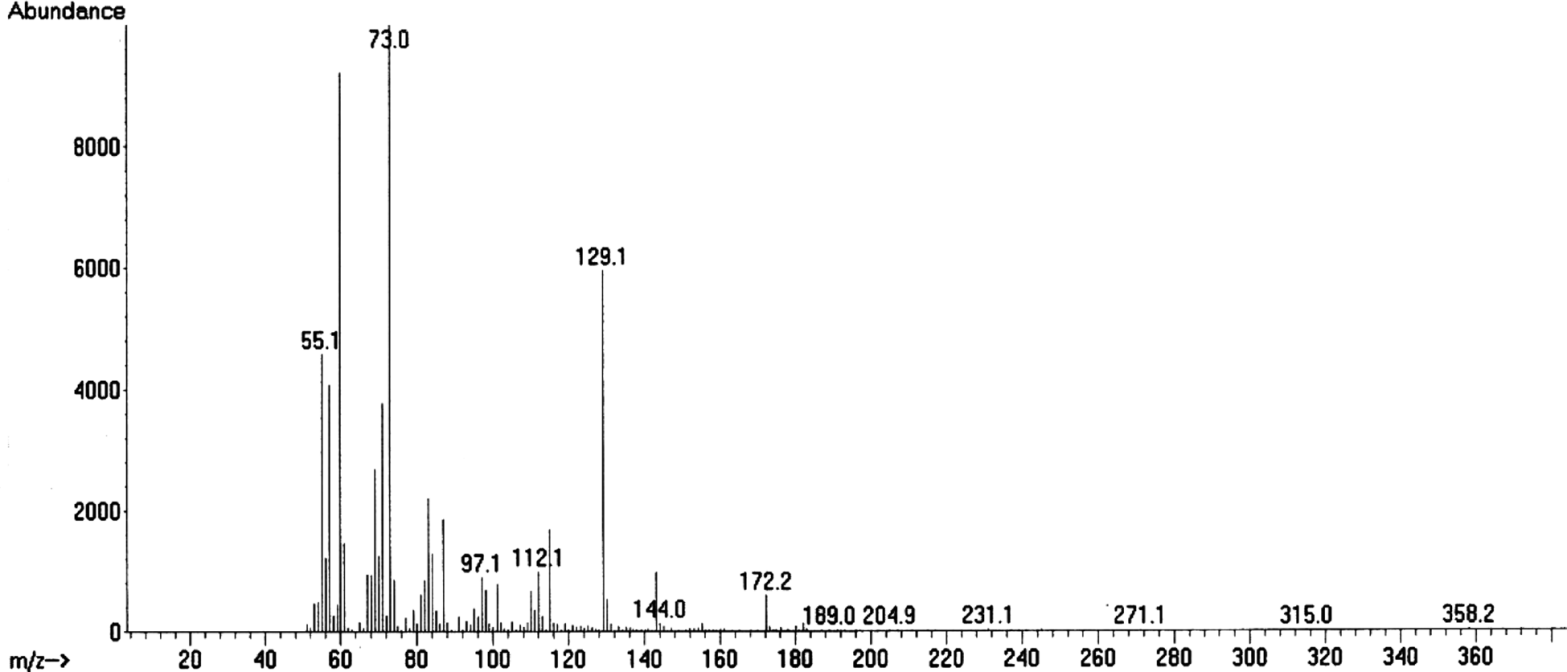
Mass spectrum of decanoic acid

**Fig. 7:**
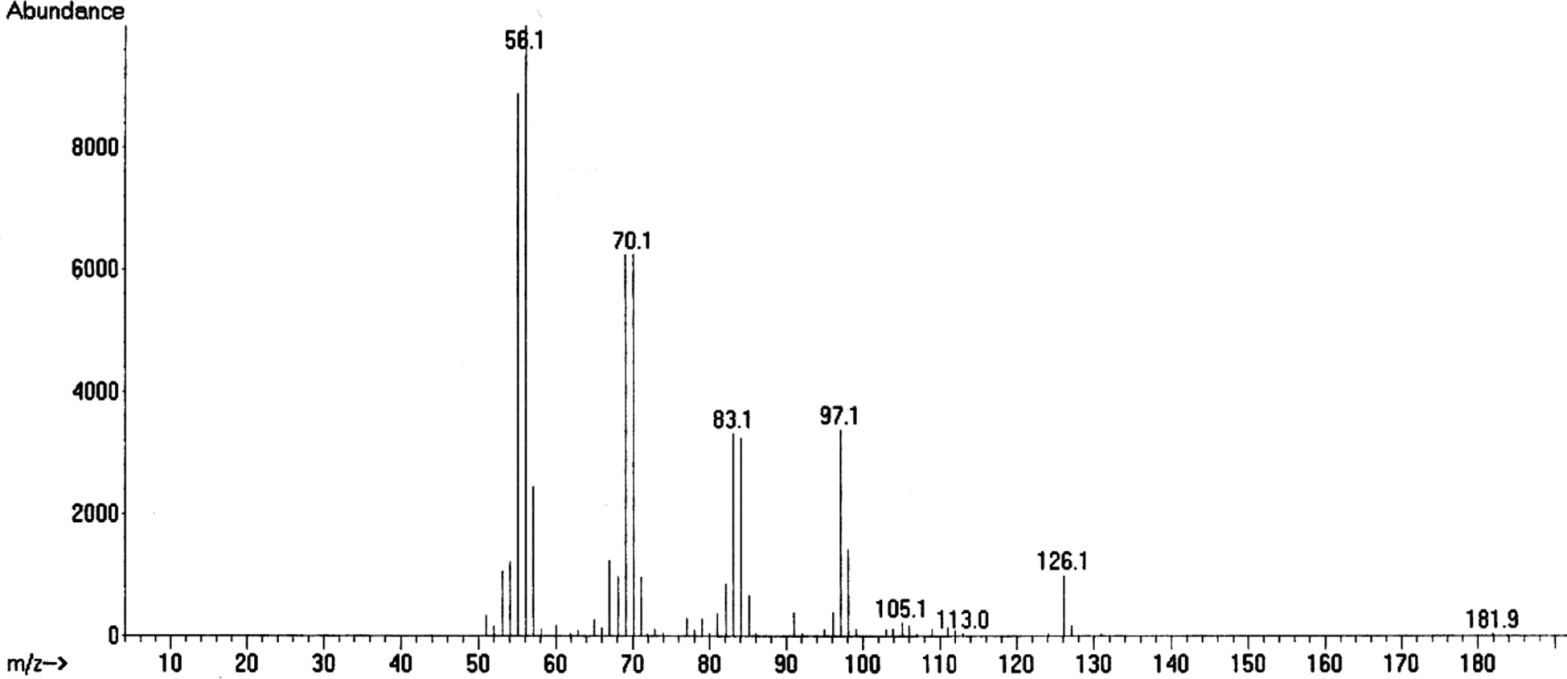
Mass spectrum of 1-nonene

 Table 1 shows the results of GC–MS analysis of the distillates D1, D2, D3 and D4 and also the organic phase of the pyrolitic oil of Na-soap of RSO. The results indicate the presence of alkanes, alkenes (nonene), cyclic compounds (cyclopentanes and cyclohexanes), aromatic compounds (toluene, butyl benzene, pentylbenzene) and carboxilic acids (heptanoic acid and decanoic acid) in the distillates of pyrolytic oil of RSO. The mass spectra of decanoic acid and 1-nonene are shown in Figs. 6 and 7 respectively.

**Table 1:** GC–MS analysis of four distillates D1, D2, D3 and D4 and the organic phase of pyrolitic oil of the sodium soap

Compound name	Formula	% total	Compound name	Formula	% total
D1 80 °C > DT			D2 80 °C < DT < 120 °C		
2-Methylpentane	C_6_H_14_	2.1	2-Methylpentane	C_6_H_14_	0.6
Methylcyclopentane	C_6_H_12_	5.4	3-Methylpentane	C_6_H_14_	0.6
Cyclohexane	C_6_H_12_	2.7	Methylcyclopentane	C_6_H_12_	8.3
*Trans*-1,2-dimethylcyclopentane	C_7_H_14_	1.2	Cyclohexane	C_6_H_12_	4.6
3-Methylheptane	C_8_H_18_	3.0	Heptene	C_7_H_14_	2.6
*Trans*-2-octene	C_8_H_16_	1.2	Methylbenzene	C_7_H_8_	1.2
*Cis*-2-octene	C_8_H_16_	19.3	*Cis*-1-ethyl-2-methylcyclopentane	C_8_H_16_	4.4
1-Nonene	C_9_H_18_	2.0	3-Octene	C_8_H_16_	1.6
1-Butylcyclopentene	C_9_H_18_	6.6	2-Octene	C_8_H_16_	0.9
1-decene	C_10_H_20_	–	1-Nonene	C_9_H_18_	1.7
Decane	C_10_H_22_	–	Decane	C_10_H_22_	2.2
D3 120 °C < DT < 140 °C					
Cyclohexane	C_6_H_12_	0.4	Heptanoic acid	C_7_H_14_O_2_	2.2
1-Heptene	C_7_H_14_	0.3	1-Undecene	C_11_H_22_	1.5
Toluene	C_7_H_8_	0.7	Undecane	C_11_H_24_	2.4
2-Octene	C_8_H_16_	1.0	2-Undecene	C_11_H_22_	2.2
1-Nonene	C_9_H_18_	3.0	5-Undecene	C_11_H_22_	0.9
*Cis*-4-nonene	C_9_H1_8_	0.9	Pentylbenzene	C_11_H_20_	1.3
1-Butylcyclopentene	C_9_H_16_	1.0	1-Dodecene	C_12_H_24_	1.4
1-Decene	C_10_H_20_	2.0	Dodecane	C_12_H_26_	2.6
Decane	C_10_H_22_	12.8	Tridecane	C_13_H_28_	0.9
4-Decene	C_10_H_20_	1.3	Pentadecane	C_15_H_32_	1.9
d-Limonene	C_10_H_16_	1.5			
Butylbenzene	C_10_H_14_	1.8			
D4 140 °C < DT < 160 °C			Na-soap of RSO		
Heptanoic acid	C_7_H_14_O_2_	5.4	1-Tetradecene	C_14_H_28_	15.7
Dodecane	C_12_H_26_	3.1	Tetradecane	C_14_H_30_	9.7
Tridecane	C_13_H_28_	3.3	Pentadecane	C_15_H_32_	12.8
Decanoic acid	C_10_H_20_O_2_	2.0	Nonoylcyclohexane	C_15_H_30_	–
Tetradecane	C_14_H_30_	4.4	3-Hexadecene	C_16_H_32_	4.1
Pentadecane	C_15_H_32_	40.2	Hexadecane	C_16_H_34_	4.2
8-Heptadecene	C_17_H_34_	7.9	Heptadecane	C_17_H_36_	3.5
Heptadecane	C_17_H_36_	15.9	Dibutylphthalate	C_16_H_22_O_4_	11.7

Nonylphenols derived from nonene are used to produce antioxidants, additives for lubricating oils, detergents and emulsifiers (Soares et al. 2008). Esters of heptanoic acid and decanoic acids are used as fragrances, artificial flavours and to esterify steroids for pharmaceutical applications (Tibor 2010; David and Milne 1993). A considerable amount of fuel based flammable hydrocarbons were also present in all four distillate fractions, and they were in the range of C_6_–C_17,_ whereas the fuel based hydrocarbons in the organic fraction of the pyrolitic oil of Na-soap were in the range of C_14_–C_16_. In the pyrolysis of soybean and palm tree oil, hydrocarbons and carboxylic acids were found to be in the range of C_7_–C_15_ whereas the hydrocarbons and carboxylic acids yielded in the pyrolysis of castor oil were in a considerably narrow range of C_15_–C_16_ and C_11_–C_12_ respectively (Lima et al. 2004). However, castor oil gave more oxygenated products such as ketones and phenols (Hanna 2011) which were not found in the pyrolysis of RSO.

The acid values, SAP values, ester values and densities of the distillates of the organic phase of pyrolytic oil are given in Table 2. The results indicate that the acid value had increased with the distillation temperature and that most of the acids were distilled out at high temperatures. This may be mainly attributed to the presence of high molecular free fatty acids and stronger intermolecular interactions in these polar carboxylic acids. Due to a similar effect, the density had also increased with the distillation temperature. The significantly high density and ester value of the RSO indicate that it has more triglycerides with strong covalent bonds. In contrast, SMOC in distillates have higher amounts of weak inter molecular interactions, and hence, low densities compared to RSO. A minor amount of ester compounds have also been noted in the distillates and this confirms the formation of small molecular esters during the pyrolysis process. This is in agreement with the ester formation step of reaction mechanisms proposed in literature (Idem et al. 1996). The presence of high molecular polar carboxylic acids was also confirmed by the results of the GC–MS analysis of the distillate fractions D3 and D4.

**Table 2:** Physical–chemical properties of different distillates

Property	ASTM method	Unit	Fraction				
			RSO	D1	D2	D3	D4
Acid value	ASTM D 664	mgKOH/g	12.0	42.9	110.1	234.1	297.3
Density at 32 °C	ASTM D4052	kg/m^3^	919.4	811.4	826.7	848.6	869.7
Saponification value	ASTM D1926	mgKOH/g	202.1	54.6	115.3	246.2	304.6
Ester value		mgKOH/g	190.1	11.7	5.2	12.1	7.3

FTIR analysis confirmed the presence of carbonyl functional groups in the distillates of pyrolitic oil of RSO. The characteristic vibration modes of aliphatic C–H stretching and C=O stretching could be identified in all four distillates at 2850–2980 cm^−1^ and around 1710 cm^−1^ respectively. The characteristic stretching vibration of carboxylic –OH was observed as a very broad band in all four spectra extending from 2400 to 3400 cm^−1^ indicating the presence of carboxylic acids. The broadness of this peak was found to be more significant when the distillates were within the high temperature range. Thus, the acidity must have increased with the distillation temperature and this confirms the increase of the acid value of distillates in the high temperature range. Also, the peaks around 1000–1300 cm^−1^ confirm the presence of ester compounds in all four fractions. The characteristic olefinic C=C stretching was observed in all the distillates except D4. This confirms that the availability of alkene content in D4 was less compared to other distillates.

## Conclusion

It has been demonstrated that RSO and its Na-soap could be converted into SMOC by using the pyrolysis technique. The results of the pyrolysis of RSO confirm the potential of producing SMOC which can be used as valuable renewable organic chemical precursors similar to petrochemicals. However, the product obtained from the pyrolysis of Na-soap consisted of more saturated alkanes of C_14_–C_17_ as found in diesel fuel. Therefore, pyrolysis of Na-soap of RSO is more suitable for producing fuel than petrochemicals. Some industrially utilised chemicals such as cyclohexane, nonene, heptanoic acid, decanoic acid and limonene were produced through the pyrolysis of RSO. The products obtained in the pyrolysis of RSO were similar to that of soybean oil and the range of products was found to be notably wider than the range of products obtained in the pyrolysis of several other fatty oils such as castor oil, palm tree oil and canola oil. The acidity and density of the distillates were found to increase with the distillation temperature. The thermal cleavage of triglycerides (TG) into free fatty acids (FFA) became significant above 150 °C and the end point of ester bonds in triglycerides was found to be at a temperature around 275 °C. The findings of this paper encourage future research in the area of pyrolysis as a renewable source for petrochemicals since currently we are currently within the declining phase of using crude oil.
